# Transcriptomics aids in uncovering the metabolic shifts and molecular machinery of *Schizochytrium limacinum* during biotransformation of hydrophobic substrates to docosahexaenoic acid

**DOI:** 10.1186/s12934-024-02381-6

**Published:** 2024-04-01

**Authors:** Iqra Mariam, Eleni Krikigianni, Chloe Rantzos, Maurizio Bettiga, Paul Christakopoulos, Ulrika Rova, Leonidas Matsakas, Alok Patel

**Affiliations:** 1 Biochemical Process Engineering, Division of Chemical Engineering, Department of Civil, Environmental, and Natural Resources Engineering, Luleå University of Technology, Luleå, SE-971 87 Sweden; 2 Department of Life Sciences – LIFE, Division of Industrial Biotechnology, Chalmers University of Technology, Gothenburg, SE-412 96 Sweden; 3 Innovation Unit, Italbiotec Srl Società Benefit, Milan, Italy

**Keywords:** Hydrophobic substrates, Docosahexaenoic acid, Thraustochytrids, Transcriptomics

## Abstract

**Background:**

Biotransformation of waste oil into value-added nutraceuticals provides a sustainable strategy. Thraustochytrids are heterotrophic marine protists and promising producers of omega (ω) fatty acids. Although the metabolic routes for the assimilation of hydrophilic carbon substrates such as glucose are known for these microbes, the mechanisms employed for the conversion of hydrophobic substrates are not well established. Here, thraustochytrid *Schizochytrium limacinum* SR21 was investigated for its ability to convert oils (commercial oils with varying fatty acid composition and waste cooking oil) into ω-3 fatty acid; docosahexaenoic acid (DHA).

**Results:**

Within 72 h SR21 consumed ~ 90% of the oils resulting in enhanced biomass (7.5 g L^− 1^) which was 2-fold higher as compared to glucose. Statistical analysis highlights C16 fatty acids as important precursors of DHA biosynthesis. Transcriptomic data indicated the upregulation of multiple lipases, predicted to possess signal peptides for secretory, membrane-anchored and cytoplasmic localization. Additionally, transcripts encoding for mitochondrial and peroxisomal β-oxidation along with acyl-carnitine transporters were abundant for oil substrates that allowed complete degradation of fatty acids to acetyl CoA. Further, low levels of oxidative biomarkers (H_2_O_2_, malondialdehyde) and antioxidants were determined for hydrophobic substrates, suggesting that SR21 efficiently mitigates the metabolic load and diverts the acetyl CoA towards energy generation and DHA accumulation.

**Conclusions:**

The findings of this study contribute to uncovering the route of assimilation of oil substrates by SR21. The thraustochytrid employs an intricate crosstalk among the extracellular and intracellular molecular machinery favoring energy generation. The conversion of hydrophobic substrates to DHA can be further improved using synthetic biology tools, thereby providing a unique platform for the sustainable recycling of waste oil substrates.

**Supplementary Information:**

The online version contains supplementary material available at 10.1186/s12934-024-02381-6.

## Background

Increased waste accumulation is one of the major environmental concerns that has emerged due to the global rise in population and has led to multifaceted endeavors for attaining environmental sustainability [1]. Waste cooking oil (WCO) and other fats generated from households, and effluents from industries are categorized under recalcitrant hydrophobic waste [2]. Considering the pernicious effects of hydrophobic waste on the environment and human health, various countries are employing strategies for its efficient recycling. The most widely accepted process is the chemical conversion of waste oil to fatty acid methyl esters for biodiesel production [3]. Compared to petrochemical processes, upcycling waste streams into value-added products via microbial fermentation could add both to sustainability and economical feasibility. Microbial populations such as yeast, fungi, bacteria and microalgae have reflected the potential to utilize hydrophobic substrates such as fatty acids and hydrocarbons. These single-celled hosts can valorize carbon derived from such waste streams into biofuel precursors and other value-added compounds [4–6].

The ω-3 fatty acid, docosahexaenoic acid (DHA) has several health benefits such as neurological and visual development in infants and prevention of cardiovascular diseases [7]. The conversion of DHA from an essential fatty acid; α-linolenic acid is very low in mammalian cells and thus it is recommended to be included in human diet. Currently, the major dietary source of DHA is fish oil, which alone fails to fulfill the increasing demand for these nutraceuticals, as fish stocks are depleting [8]. Thraustochytrids have gained attention as a major source of very long chain ω-3 and ω-6 PUFAs; eicosapentaenoic acid (EPA), DHA and docosapentaenoic acid (DPA) [9]. Specifically, *Schizochytrium limacinum*, *Aurantiochytrium mangroovi* and *Parietichytrium* sp. can accumulate DHA up to 50% of total fatty acids [10–12]. Thraustochytrids have been extensively investigated for their ability to assimilate and convert glucose and other hydrophilic carbon substrates into DHA [13]. Given their ability to grow in a variety of carbon sources and tolerate high salinity, thraustochytrids have recently been studied by Patel et al. for their potential to utilize hydrophobic waste oil [4]. Considering the high-market value of ω-3 fatty acids, valorization of these waste streams into DHA by thraustochytrids is advantageous over yeast-derived biodiesel precursors.

Microbial populations employ varying metabolic routes for the biotransformation of these hydrophobic waste. In the majority of microbes, secretory lipases hydrolyze triacylglycerides (TAGs) into free fatty acids and glycerol, thereby enabling them to utilize oil as a sole carbon source. Genomic analysis has identified 25 putative extracellular and intracellular lipases in *Y. lipolytica* [14, 15]. Prasad et al. reported that hydrocarbonoclastic bacteria *Alcanivorax borkumensis* forms dendritic biofilms that have tubulation which allows oil assimilation [16]. Further, these fatty acids are transported across the plasma membrane either via fatty acid transporters (*fat*), passive diffusion, or through endocytosis [17–20]. The imported fatty acids are then activated by acyl CoA synthetases (ACS) for either entering β-oxidation pathway for generating acetyl-CoA or incorporating into TAG molecules [21]. Previous studies have identified the physiological effects of these substrates using kinetic models in *Y. lipolytica* [22, 23]. Several hypotheses have been proposed regarding the metabolic fate of these substrates, however relevant omics datasets are scarce. Thus, till date, the complete assimilation route for fatty acids is not universally agreed upon.

**Fig. 1:**
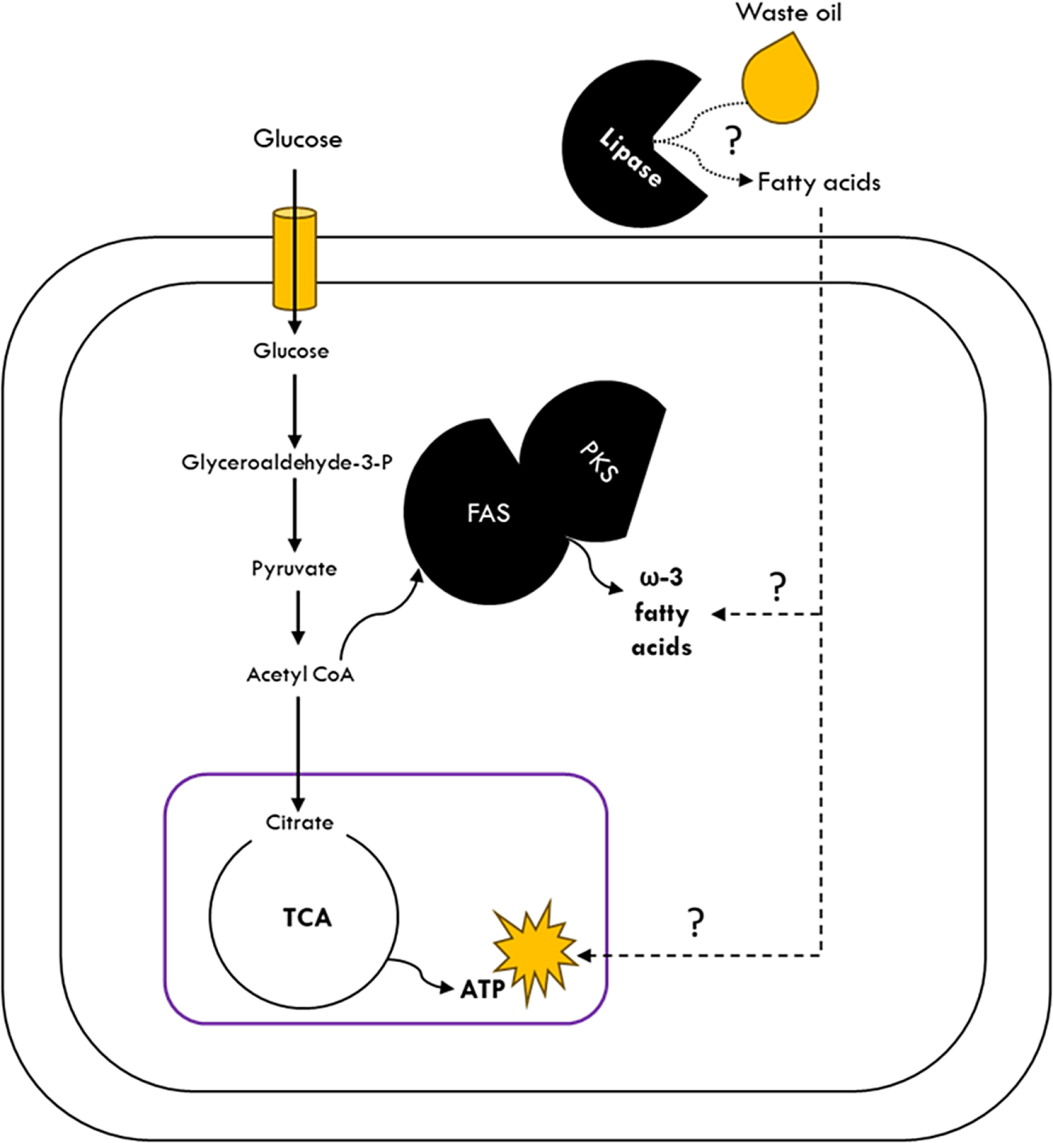
Schematic illustration depicting the “*de novo*” and unidentified route of “*ex novo*” lipid synthesis. Solid arrow depicts lipid synthesis in the presence of glucose and dashed arrows depicts lipid synthesis in the presence of hydrophobic substrates

Thraustochytrids have a unique blend of both eukaryotic and prokaryotic pathways, making them extremely interesting to study [10, 24, 25]. They possess polyketide synthase (PKS) clusters for polyunsaturated fatty acids (PUFAs) production, which has been reported for marine bacterial species such as *Shewanella* sp. and *Moritella marina* [26]. Previously, transcriptomics and metabolomics have been extensively employed to understand how glucose and non-glucose substrates such as glycerol are converted to ω-3 fatty acids i.e., through *de novo* lipid synthesis [27, 28]. However, there is limited knowledge about how thraustochytrids uptake lipids from the environment and what molecular machinery is involved in this process. Ishibashi et al. reported a secretory lipase in *Aurantiochytrium limacinum* which suggests its ability to utilize TAG substrates [11]. Yet, there is no evidence if the released fatty acids are imported actively and how these are converted into ω-3 fatty acids (Fig. 1). Conversion of these waste lipid substrates to high-value intracellular lipids i.e., DHA is hereafter referred to as “*ex-novo*” lipid synthesis.

Here, we aimed to identify the metabolic route for waste oil assimilation and its conversion to DHA in marine thraustochytrid *Schizochytrium limacinum* SR21. Primarily, the biodegradation potential of SR21, along with its ability to upgrade the lipid composition of the hydrophobic substrates (HS) towards the production of microbial lipids rich in nutritional fatty acids was investigated. For this purpose, SR21 was grown on a variety of commercial and waste-derived oils with different fatty acid composition as the sole carbon source. The thraustochytrid was able to efficiently valorize HS into intracellular DHA, along with enhanced biomass yield as compared to glucose. Further, transcriptomic analysis was performed for WCO2 (waste cooking oil #Lot 2), mCOs (mixture of commercial oils) and compared with glucose, to trace the metabolic pathways involved in the utilization of HS. The RNAseq data highlights the upregulation of transcripts encoding for both secretory as well as intracellular lipases that convert TAG into free fatty acids. Further, the transcriptomic datasets reveal that these oil substrates are assimilated via endocytosis, peroxisomal and mitochondrial β-oxidation to generate acetyl-CoA. In comparison to glucose, the cells cultivated in WCO2 and mCOs were found to cope with the oxidative stress, suggesting that SR21 can thrive effortlessly in a hydrophobic environment.

## Methods

### Strain and growth conditions

*Schizochytrium limacinum* (ATCC-MYA-1381, SR21) strain was obtained from the American Type Culture Collection (ATCC, USA). The strain was maintained in medium composed of 20 g L^− 1^ glucose, 7.2731 g L^− 1^ yeast extract to regulate the C/N ratio at a value of 10, the salinity level was set at 9 ppt by diluting accordingly artificial seawater (ASW) and the pH was adjusted at 6.8. The composition of ASW (18 ppt salinity) was: NaCl (0.31 M), MgSO_4_⋅7H_2_O (10.5 mM), KCl (8 mM), NaNO_3_ (11.8 mM), CaCl_2_⋅2H_2_O (2 mM), KH_2_PO_4_ (0.37 mM), Tris buffer (25 mM), NH_4_Cl (0.5 mM), vitamin B_12_ (0.1 µM), chelated iron solution (1 mL L^− 1^) containing Na_2_EDTA⋅2H_2_O (26 µM), 0.1 M HCl (45 mM), FeCl_3_⋅6H_2_O (3 µM) and metal solution (10 ml L^− 1^) containing Na_2_EDTA⋅2H_2_O (2.7 mM), H_3_BO_3_ (18.4 mM), FeCl_3_⋅6H_2_O (0.18 mM), MnSO_4_⋅H_2_O (0.97 mM), ZnSO_4_⋅7H_2_O (0.07 mM), CoCl_2_⋅6H_2_O (0.02 mM). A 10% v/v inoculum was added to the culture media to reach a final volume of 100 mL in 250 mL Erlenmeyer flasks. The culture was incubated at 25 ^o^C and 180 rpm for 48 h before inoculation to the hydrophobic media.

### Cultivation of SR21 on hydrophobic substrates (HS)

The hydrophobic media (HM) consisted of 18 ppt salinity ASW, 10 g L^− 1^ of carbon source, yeast extract was added accordingly to regulate the C/N ratio at a final value of 10 and the pH was adjusted to 6.8. The carbon sources used in the HM preparation were the following: glucose was used as a reference, commercial oils (Olive, Sunflower, Peanut, Flaxseed, Fish and mixture of the five commercial oils in equal parts (mCOs)) and two different types of waste cooking oils (WCO1 and WCO2). WCO1 was obtained from the local grocery store nearby the university campus, while WCO2 was received from a university restaurant. For inoculation, 10% v/v of preculture cells was centrifuged (8000 rpm, 4 ^o^C for 10 min) and was then added to HM. The cells were cultivated for 72 h at 25 ^o^C and 180 rpm. To test the optimal carbon source concentration, cultivation of the strain was carried out in HM containing mCOs in different concentrations (10, 20, 40, 80 g L^− 1^) in addition to yeast extract as the nitrogen source in appropriate amounts to maintain a C/N ratio of 10. Harvesting of the cells was carried out after 10 days of cultivation.

### Substrate consumption and dry cell weight estimation

Cells were harvested by centrifugation (8000 rpm, 4 ^o^C for 10 min), and the supernatant was collected for residual substrate quantification. To determine the residual oil present in the supernatant of the HM, 10 mL of n-hexane was added and kept for approximately 1 h under shaking conditions. The mixture was then centrifuged and the upper phase containing the residual oil was transferred to a glass vial. The residual oil was measured gravimetrically after drying in a hot air oven. Cell pellets were washed with distilled water, lyophilized and measured gravimetrically for dry cell weight (DCW) estimation and expressed in g L^− 1^ of culture. Residual glucose in the supernatant was determined using high-performance liquid chromatography (HPLC) as previously described [4].

### Lipid extraction and GC-MS analysis

Total lipids were extracted using the method by Bligh and Dyer [29]. Specifically, chloroform: methanol (2:1 v/v) was mixed with the mortar-pestle pulverized freeze-dried biomass. After overnight shaking at room temperature (RT), the mixture was centrifuged, and the supernatant was filtered using a PTFE (0.2 μm) filter into glass vials and left in a 55 ^o^C hot-air oven until constant weight. The total lipids were estimated gravimetrically and expressed in g L^− 1^. For GC-MS analysis, the lipids (intracellular and substrate) were transesterified as mentioned by Wychen et al. [30]. For transesterification, 1 mL chloroform: methanol (2:1 v/v) was added to ~ 50 mg of lipids, and transferred into an ace pressure glass tube. Acid catalyst, 2 mL of 0.6 M methanolic HCl solution was added, and the mixture was incubated at 90 ^o^C for 1 h. After cooling, n-hexane (1 mL) was mixed for the extraction of fatty acid methyl esters (FAMEs) and 1 mL of water was added to separate two phases. After centrifugation, the upper layer containing the FAMEs was analyzed using gas chromatography-mass spectrometry (GC-MS) (Clarus 690 MS coupled to a Clarus SQ8 GC, PerkinElmer, Waltham, MA, USA) equipped with a capillary column (Elite -FFAP; 30 m, 0.25 mm ID, 0.25 μm df, Cat. # N9316352; PerkinElmer). The analysis was performed as described [4].

### Fractionation of lipids

Total lipids were fractionated into neutral lipids (NLs), glycolipids (GLs) and phospholipids (PLs) using a silica cartridge Sep-Pack of 1000 mg according to Berge et al. [31]. Column conditioning was performed using 30 mL chloroform. Approximately 20 mg of lipids in 1 mL of chloroform was loaded, followed by addition of 15 mL chloroform: acetic acid (9:1 v/v) for the elution of NLs. The GLs were eluted after addition of 20 mL acetone: methanol (9:1 v/v) and PLs after addition of 20 mL methanol. Each fraction was separately collected and air-dried in a 50 ^o^C oven and measured gravimetrically.

### RNA extraction, library preparation and RNA-sequencing

For transcriptome analysis, SR21 was cultivated in glucose, WCO2 and mCOs with substrate concentration of 20 g L^− 1^ and a C/N ratio of 10. Cells were collected at 72 h, was snap-freezed and stored at – 80 °C. TRIzol reagent was used for extraction of RNA as described by Rio et al. [32]. Briefly, 1 mL of TRIzol reagent was added to the quenched cells and vortexed for 10 min. Samples were incubated at RT for an additional 2–3 min followed by 10 min centrifugation at 13000 rpm (4 ^o^C). The obtained supernatant was transferred to an eppendroff and 250 µL of chloroform was added and mixed vigorously, followed by vortex for 2 min. The upper layer was collected after centrifugation at 13000 rpm for 15 min (4 ^o^C). For RNA precipitation, chilled isopropanol was added to the aqueous phase in equal proportion and the mixture was incubated for 30 min on ice. RNA precipitate was obtained by centrifugation at 13000 rpm for 10 min. To remove salts and phenolic contaminants, pellets were washed twice with 70% ethanol. Total RNA was eluted in 50 µL of RNAse-free water. Enrichment of mRNA was done using oligo dT beads that bind to the polyA tail. First-strand cDNAs were generated from fragmented mRNA using N6-primed reverse transcription, followed by the synthesis of second-strand of cDNA. cDNA was further end-repaired, 3’ adenylated and ligated to adaptors. Using primers, PCR amplification was carried out to enhance the purified cDNA template. After pooling, the DNA double strands were heat-denatured into single strands, and the ligase and cyclic buffer were added to create DNA circles through the cyclization step. Rolling circle replication (RCR) was utilized to create DNBs using DNA circles. Sequencing was performed using the BGI-DNBseq^TM^ platform.

### Differential gene expression analysis

Raw reads were filtered using BGI software SOAPnuke [33] and the clean reads were mapped to the available genome of *S. limacinum* SR21 available at NGDC (Accession no. GWHABLD00000000) using HISAT2 [34]. Novel transcripts were predicted using StringTie [35], Cufflinks tool [36]. The coding potential of novel transcripts was predicted using CPC tool [37]. Clean reads were mapped to reference using Bowtie2 [38], and then gene expression level was estimated using RSEM [39]. DESeq2 [40] was used for differential expression analysis by applying Wald’s test, and the Benjamini and Hochberg method [41] was utilized to adjust the p-values that were obtained. DEGs with fold Change > = 2.00 and adjusted p-value < = 0.05 were considered significant.

### Functional annotation of DEGs

The DEGs were further classified into GO categories: Biological Process, Molecular Function, and Cellular Component using eggNOG-mapper [42]. Further, the DEGs were also assigned with the KEGG IDs using BlastKOALA [43]. Domain analysis for identification of lipases was performed using InterProScan [44]. For subcellular localization, WoLFPSORT [45] and DeepLOC 2.0 [46] were employed. SignalP 5.0 [47] was used for prediction of secretory signals whereas TMHMM 2.0 [48] was used for prediction of transmembrane domain in the protein. Transcription factors were predicted using PlantTFDB 4.0 [49] while carbohydrate activating enzymes (CAZymes) are predicted using dbCAN [50].

### qPCR validation of significant transcripts

The qPCR analysis was performed for few relevant transcripts. TRIzol reagent was used to extract RNA as described previously, and RevertAid First Strand cDNA Synthesis Kit (Thermo Scientific, USA) was used to synthesize cDNA from 1 µg of total RNA using oligodT primers according to the manufacturer’s procedure. Using PCR ABI 7900HT and PowerTrack™ SYBR™ Green Master Mix (BIO-RAD), real-time qPCR was performed. The housekeeping gene, 18 S rRNA of SR21 was used as an internal reference to analyze the quantitative variation using the relative quantitative approach (ΔC_t_) [51]. The primers used for qPCR are listed in Supplementary_file 1: Table 1.

### Hydrogen peroxide (H_2_O_2_) content

For H_2_O_2_ estimation, freeze-dried biomass (25 mg) was pulverized with 2 mL of 0.1% w/v trichloroacetic acid (TCA) followed by overnight incubation at RT with shaking. The solution was centrifuged (10000 rpm, 4 ^o^C for 10 min) and the supernatant was filtered through RC (0.2 μm) filter. The supernatant was used for the determination of H_2_O_2_ content as described by Velikova et al. [52]. Briefly, 0.5 mL of 0.01 M phosphate buffer (pH 7.0) was mixed with equal proportion of supernatant and 1 mL of 1 M potassium iodide (KI). Control samples containing water instead of KI were also prepared for every sample to correct the background signal. 200 µl of the reaction mixture was transferred to a 96-well UV-microplate and the absorbance was measured at 390 nm after 10 min of incubation at RT under dark conditions. For quantification of H_2_O_2_ content, a calibration curve with H_2_O_2_ standard solutions prepared in 0.1% w/v TCA was used and was expressed in nmol g^− 1^ dried biomass.

### Malondialdehyde (MDA) assay

The peroxidation level of lipids was determined by performing the MDA or thiobarbituric acid-reactive-substances (TBARS) assay. 25 mg of freeze-dried biomass was extracted with 2 mL of 5% w/v TCA and was incubated overnight at RT with shaking. The supernatant collected after centrifugation (10000 rpm, 4 ^o^C for 10 min) was filtered through RC (0.2 μm) filters, followed by estimation of the MDA equivalents as described by Heath and Packer [53]. Briefly, 1 mL of the supernatant was added into ace pressure glass tubes together with 1 mL of 0.65% thiobarbituric acid (TBA) which was prepared in TCA (20% w/v). The mixture was heated to 95 ^o^C for 25 min and was centrifuged after reaching an ambient temperature. The absorbance of the supernatant was measured at 600 and 532 nm wavelengths and the MDA equivalent content (nmol ml^− 1^) was estimated using the following equation, based on the extinction coefficient of 155 mM^− 1^ cm^− 1^ and was finally expressed in µmol g^− 1^ dried biomass.

$$\begin{aligned}&\text{MDA}\;\text{equivalents}\;(\text{nmol}\cdot \text{ml}^{-1})\end{aligned}$$

$$=\left[\right({\text{A}}_{532 \text{n}\text{m}}-{\text{A}}_{600 \text{n}\text{m}})\cdot {10}^{6}]/155000$$

### Total flavonoid content (TFC) and total phenolic content (TPC) quantification

Freeze-dried biomass (25 mg) was homogenized with 2 mL of 95% v/v methanol and was incubated overnight at RT with shaking. After centrifugation (10000 rpm, 4 ^o^C for 10 min) of the homogenate, the supernatant was filtered as described previously and the TFC and TPC content was measured. The TFC was determined using the method described by Gomez et al., with minor modifications [54]. Briefly, 1 mL of the supernatant was mixed with 4 mL of H_2_O and 0.3 mL of 5% w/v NaNO_3_, and after 6 min incubation, 0.3 mL of 10% w/v AlCl_3_ was added. After another 5 min, 2 mL NaOH was added and volume was adjusted to 10 mL using H_2_O. The absorbance of the solution was measured at 415 nm and was corrected by the absorbance of a sample blank containing methanol instead of cell extract. A calibration curve with standard solutions of quercetin (QE) prepared in 95% v/v methanol was used for quantification and the TFC levels were expressed as mg QE g^− 1^ dried biomass. The TPC was estimated by using the method by Singleton et al., with some modifications [55]. Briefly, 0.5 mL of supernatant was mixed with 0.5 mL of 1 N Folic Ciocalteu (FCR) reagent and 1 mL of 20 g L^− 1^ Na_2_CO_3_. Finally, H_2_O was added to a final volume of 5 mL and the mixture was incubated for 90 min at 50 ^o^C. After cooling, the mixture was centrifuged, and the absorbance of the extracts was measured at 760 nm against a blank sample containing methanol instead of cell extract. Gallic acid (GA) standard solutions prepared in 95% v/v methanol were used for the calibration curve and the TPC levels were expressed as mg GA g^− 1^ dried biomass.

### Determination of reduced and oxidized glutathione levels in cells

Total and oxidized glutathione (GSH) in fresh cells harvested after 72 h of cultivation was measured using the method described by Rahman et al. [56]. Specifically, the cells were homogenized with 1 mL extraction buffer, followed by centrifugation (8000 rpm, 4^o^C for 10 min). The cell extracts were then assayed for their total GSH content as follows: 20 µl of appropriately diluted extract was mixed with 120 µl dithionitrobenzoic acid: glutathione reductase (DTNB: GR) solution. After 30 s, 60 µl of β-NADPH was added and measurements of the absorbance at 412 nm were taken, every 30 s for 2 min. Together with cell extracts, a sample blank and glutathione standards were measured to create the calibration curve for GSH. The rate of TNB formation was calculated as change in the absorbance (ΔA min^− 1^) and the calibration curve for GSH was used for quantification of the total GSH present in the samples and expressed as nM mg^− 1^ biomass. For the oxidized glutathione (GSSG) estimation, a derivatization step is necessary using 4-vinylpyridine (4VP) which covalently reacts with GSH. Thus, 2 µl of 4VP was mixed with 100 µl of cell extract and kept at RT for 1 h. After addition of 6 µl triethanolamine and 10 min incubation, the sample was assayed as previously described. A sample blank and GSSG standards were also included in the derivatization step. All the samples were assayed as previously described and the GSSG concentration in the samples was expressed as nM mg^− 1^ biomass. The concentration for GSH was calculated using the following equation [GSH]= [GSH_total_]-2×[GSSG] and the GSH/GSSG ratio was estimated.

### Statistical analysis

Apart from the transcriptome analysis, Principal Component Analysis (PCA) and Spearman’s correlation analyses were performed using Origin Pro 2023 software (Origin Lab Corporation, Northampton, MA, USA).

## Results and discussion

### Valorization of hydrophobic substrates by thraustochytrid SR21

**Fig. 2:**
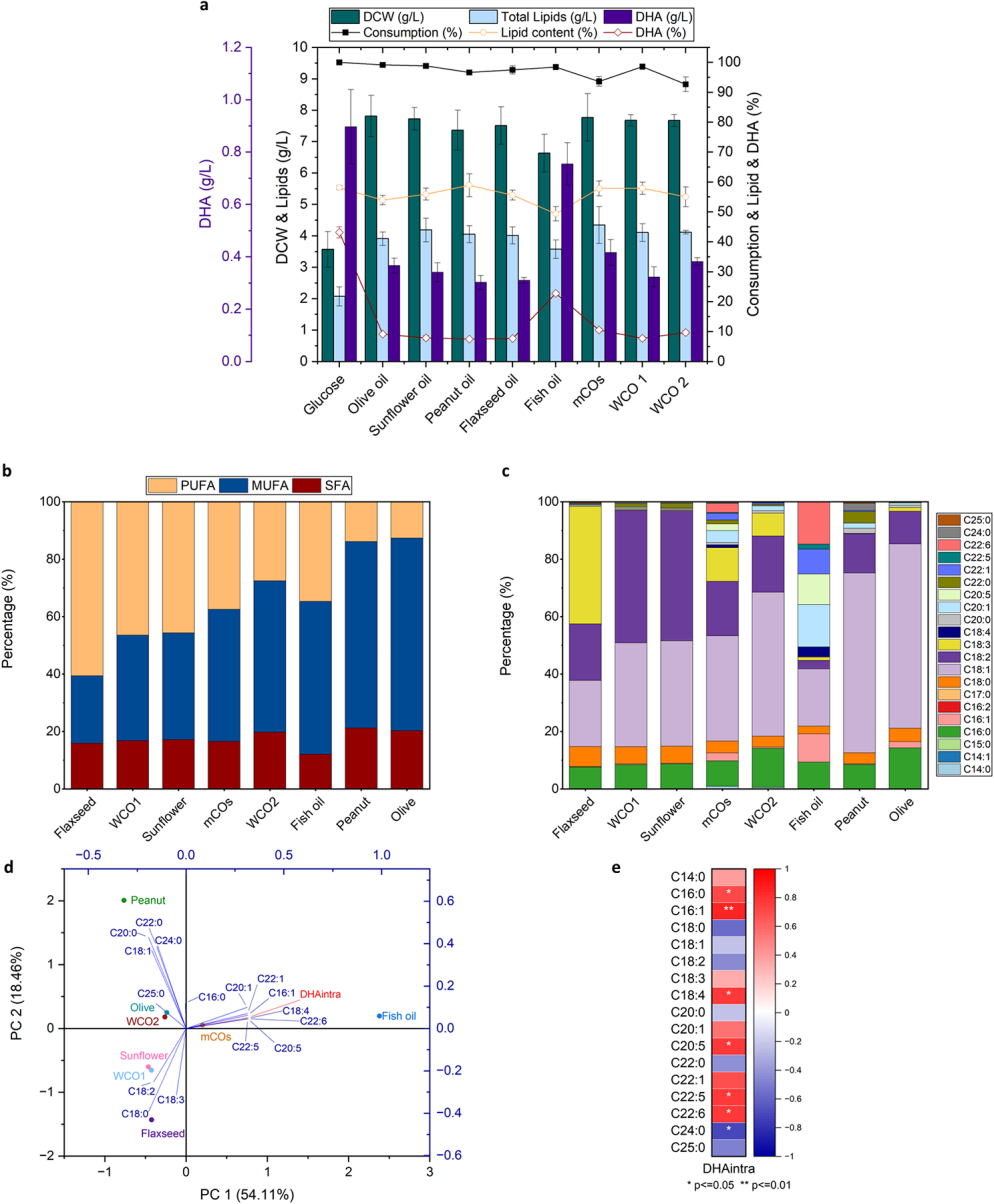
Shake flask cultivation of SR21 in 10 g L^− 1^ hydrophobic substrates (HS) and glucose (72 h). **(a)** Growth-associated parameters (substrate consumption (%), dry cell weight (DCW; g L^− 1^)), lipid and DHA (% and g L^− 1^) are shown. The indicated values represent the mean and s.d of three biological replicates; **(b)** Saturation profile i.e., saturated fatty acid (SFA), monounsaturated fatty acid (MUFA) and polyunsaturated fatty acid (PUFA) content of HS; **(c)** Fatty acid profile of HS analyzed by gas chromatography-mass spectrometry (GC-MS); **(d)** Principal Component Analysis (PCA) of the HS fatty acids and intracellular DHA (DHAintra). The PCA biplot is visualizing the HS as scores of different colors and their fatty acid profile as loading factors of blue color. Intracellular DHA (DHAintra) is shown as a loading factor of red color. A total of 72.57% variance of the dataset is explained by principal component (PC) 1 and 2, where PC1 accounts for 54.1% and PC2 for 18.46% of the variance; **(e)** Correlation map using Spearman’s correlation analysis between DHAintra and each of the fatty acids present in all HS used in this study. Spearman’s rho (r) is illustrated as color variation, where red color indicates a positive r-value and blue a negative r-value. The asterisks denote statistical significance * (p < = 0.05), ** (p < = 0.01) of the correlation between the variables examined

In this study, to obtain a better understanding of the metabolic fate of SR21’s growth patterns, the thraustochytrid was grown on a variety of HS with different fatty acid profiles The initial concentration of the carbon source was 10 g L^− 1^ and glucose was used as the reference condition. Different cultivation parameters, such as biomass (DCW; g L^− 1^), substrate consumption (%), as well as lipid and DHA (concentration; g L^− 1^ and content; % DCW) were determined as depicted in Fig. 2a. As shown, SR21 could efficiently consume more than 90% of all oils within 72 h. The biomass yield obtained using HS was 7.5 ± 0.4 g L^− 1^ (average of all HS), which was 2-fold higher as compared to glucose. A similar trend of improved biomass was observed for strains of *Schizochytrium* species when grown on glucose in combination with HS, such as waste cooking oil and flaxseed oil [4, 57]. In the past decade, thraustochytrids have been extensively optimized for DHA production by modulating cultivation parameters, such as carbon source, concentration, oxygen levels, temperature and salinity. Hydrophilic carbon substrates were previously employed, although there are only a few studies related to HS consumption [4, 57–59]. Yet, the effect of the specific lipid profile or which individual fatty acids significantly alter the physiology of thraustochytrids is not studied till date.

The lipid content was relatively similar between HS (55.6 ± 2.8%, average of HS) and glucose (58.1 ± 0.7%), however, the total lipids yields were 2-fold higher in HS due to increased lipid-free biomass. Notably, the DHA content was significantly higher (5-fold) when the strain was cultivated in glucose, reaching 43.2% of total lipids as compared to HS (8.6 ± 1.1% average of HS). This is in agreement with previous studies, indicating that compared to glucose, HS promote relatively less DHA synthesis when used as the sole carbon source [4, 57–59]. The enhanced lipid-free biomass obtained through the utilization of HS reduced the difference between the achieved DHA yields in comparison to glucose. Elevated cell growth in a hydrophobic environment accompanied by lower DHA biosynthesis suggests the regulated distribution of acetyl-CoA precursors between the TCA cycle and PKS clusters [27]. Among the HS, fish oil resulted in the highest intracellular DHA content which accounted for 22.8% of the total lipids. After fish oil, the maximum DHA content of the intracellular lipids was obtained by mCOs followed by WCO2 and olive oil (10.5, 9.7 and 9.3%, respectively), while the lowest content was observed for peanut oil (7.5%). To figure out the existence of a reasonable basis behind the observed trend it is important to investigate the variations of the HS lipid profiles. It should be highlighted that fish oil has a unique and more complex lipid profile compared to the other oils, which includes DHA (Fig. 2c), therefore not all the DHA measured in the intracellular lipids is synthesized *de novo* but is likely originating from the unhydrolyzed DHA present as free fatty acid within the cell.

Further, to evaluate the effect of increasing carbon source concentration, SR21 was cultivated in the range of 10–80 g L^− 1^ of mCOs as substrate. By observing the acquired growth results and the DHA yields, as shown in Supplementary_file 1: Fig. 1, it is noted that the optimal concentration for SR21 was 20 g L^− 1^, as a further increase in the concentration resulted in lower intracellular DHA content.

### C16:1 and C16:0 positively affect intracellular DHA accumulation

To comprehend the effect of the oil composition on the “*ex novo*” lipid synthesis pathway and particularly whether DHA biosynthesis is differentially activated depending on the substrate, statistical techniques, such as principal component analysis (PCA) and Spearman’s correlation were employed. The saturation profile i.e., saturated (SFA), monounsaturated (MUFA) and polyunsaturated (PUFA) fatty acid percentages of each of the oils is shown in Fig. 2b and their specific fatty acid (FA) profile is shown in Fig. 2c. The PCA biplot is depicted in Fig. 2d, where the oils used as substrates are presented as scores and their FA profile as factor loadings. Comparison of the location of the loading factors with the scores in the PCA biplot is indicative of the distinctions among the oils and their FA composition. As expected, the scores for sunflower oil and waste cooking oil 1 (WCO1) are positioned closely in the negative quadrant in the PCA biplot since they have similar FA profiles, with an average PUFA level of 46.1% (± 0.4%) of total lipids which is composed mostly of C18:2. Olive oil and WCO2 are both rich in C18:1, C18:2 and C16:0, but WCO2 contains higher levels of C18:3 compared to olive oil. The scores for flaxseed, peanut and fish oil are in different quadrants indicating significant differences in their FA composition. Fish oil is rich in LC-PUFAs, specifically C22:6, followed by C20:5 and C22:5 and LC-MUFAs like C20:1 and C22:1. It contributes to the LC-PUFAs present in mCOs in lower amounts, thereby mCOs has a unique composition of both LC-PUFAs and C18 PUFAs and would be an ideal substrate for understanding its physiological significance. The dominant type of FAs in mCOs was MUFAs (46% of total lipids), mainly C18:1, followed by PUFAs (37.5% of total lipids) consisting mostly of C18:2 and C18:3.

Identification of possible correlation among the intracellular DHA (DHAintra) with the distinctive substrate FAs is possible by comparing the cosine of the angle between the factor loadings which indicates the direction and the strength of the correlation, so that if the angle is small there is a strong positive correlation, if the angle is 90^o^ there is no correlation, whereas if the angle is above 90^o^ there is a negative correlation [60]. In PCA biplot, the loading factors closest to DHAintra are the ones that belong to the FAs present in higher amounts in fish oil, followed by mCOs. All of them are MUFAs and PUFAs with more than 20 carbon atoms, except for C16:1. Furthermore, it is noted that the FAs present in the upper left quadrant, like C18:1 and LC-SFAs, form an angle close to 90^o^ with DHAintra, therefore no significant correlation can be assumed between them. On the other hand, the three FAs (C18:0, C18:2 and C18:3) located in the negative quadrant indicate a negative correlation with DHAintra. The loading for C16:0 can potentially have a positive weak correlation with DHAintra.

These observations are further supported by the Spearman’s correlation coefficients, illustrated in the heatmap in Fig. 2e. The intracellular DHA was found to have the highest positive correlation with C16:1 (*r* = 0.857, p-value = 0.007) and C16:0 (*r* = 0.714, p-value = 0.047). Also, a positive correlation was observed with FAs C18:4, C20:5, C22:1, C22:5 and C22:6 (*r* = 0.764, p-value = 0.027), however, these are only present in fish oil and mCOs and thus might provide a biased correlation with DHAintra, further highlighting the significance of C16:1 and C16:0 as the FAs with the strongest positive correlation with intracellular DHA. A similar analysis to understand how the oil composition influences cell growth failed to provide meaningful insight, since no significant differences were found among the DCW values within the substrates tested. Papanikolaou et al. also observed that after cultivating *Y. lipolytica* in HS of varying FA composition, the cell growth didn’t differ significantly, whereas lipid accumulation was favored by stearin-rich media, enriched in C18:0^23^. Furthermore, the same yeast strain showed increased specificity towards certain FAs, where MUFAs, like C18:1 rapidly accumulated for growth-associated purposes, while SFAs, such as C16:0 and C18:0 had lower assimilation rates but were mostly used for the accumulation of storage lipids [22, 61].

DHA synthesis in SR21 occurs via the PKS pathway, which uses acetyl-CoA as precursor. These statistical tests highlight that intracellular DHA is positively affected by C16:0 and C16:1 fatty acid content in HS, suggesting their efficient conversion into acetyl-CoA generated from the β-oxidation pathway for the “*ex novo*” fermentation. As mentioned previously, ACS is the key enzyme required for FA activation before entering the β-oxidation cycle. This has a distinct substrate specificity, which is directly influenced by the length of the carbon chain or the degree of unsaturation. It can be therefore assumed that for SR21 the ACS might have a preference specifically for C16 FAs. Also, C16:1 showed stronger correlation and statistical significance than C16:0, indicating a promising potential substrate for ACS. Thus, C16:0 or C16:1 might be the only precursors able to start the energy production and lipid biosynthesis in SR21.

### “*De novo”* lipid synthesis yields higher DHA

**Fig. 3:**
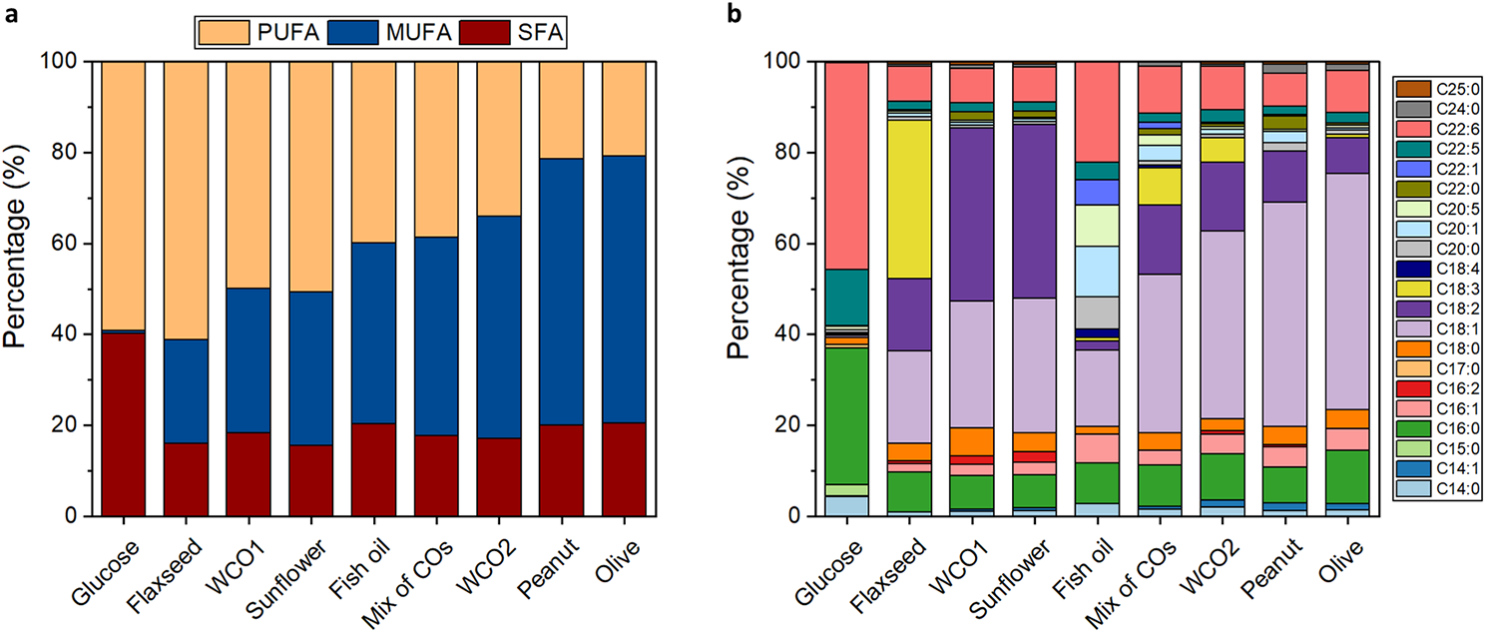
Intracellular lipids composition of SR21 cultivated in 10 g L^− 1^ HS and glucose. **(a)** Saturation profile of SR21 lipids, in terms of SFA, MUFA and PUFA content; **(b)** Fatty acid profile of SR21 lipids analyzed by GC-MS.

Figure 3a, depicts the profile of intracellular lipids in terms of saturation, while Fig. 3b displays the FA composition of SR21 cultivated in oils and glucose. As shown, the lipids produced in the presence of glucose contained mainly PUFAs and SFAs, while no significant amounts of MUFAs were observed. The PUFAs were rich in DHA which consisted of 45.5% of the total FAs, followed by 12.3% DPA, 0.8% EPA and minor levels of C18 MUFAs and PUFAs. In contrast, the lipids produced when SR21 was grown on HS had much lower levels of DHA ranging from 7.3% (peanut oil) to 22.2% (fish oil), while the DPA content varied from 1.8 to 3.9%. Compared to glucose as a carbon source, fish oil and mCOs were found to have 11 and 2.9-fold higher EPA content respectively, whereas for other HS it was relatively lower. These observations are in agreement with previous studies where the DHA content was lower for HS compared to glucose. Specifically, the reported intracellular DHA content reached values up to 30% of total FA in glucose-rich media, whereas it was ~ 10% when thraustochytrids were grown in varying HS, like linseed oil, C18 FAs, waste acid oil and waste cooking oil [4, 58–60].

Except for PUFAs synthesized by the thraustochytrid strain *de novo* through the PKS pathway, the FAs C14:1 and C16:2 were detected in most of the SR21 lipids, without being present in the hydrophobic substrates. Besides that, the FA profile of SR21 was very similar to that of the respective oil substrate, with a reduced content of C18 FAs. The most abundant SFA in glucose-induced lipids was C16:0, whereas its content was reduced in the presence of HS. On the contrary, C14:0, C14:1, C16:1 and C16:2 content was increased in SR21 cultivated in HS. The increase of C16:2 content in SR21, indicates that the cell contains desaturases responsible for the conversion of C16:1 to C16:2. However, this was only observed for the case of HS, since no amounts of C16:1, nor C16:2 were found in SR21 cultivated in glucose. It is therefore assumed that during the “*ex novo*” and “de novo” cell growth different enzymes are activated which promote the cellular functions towards growth and lipid synthesis, accounting for the differences in the intracellular lipid composition of SR21.

### Transcriptomic analysis

**Fig. 4:**
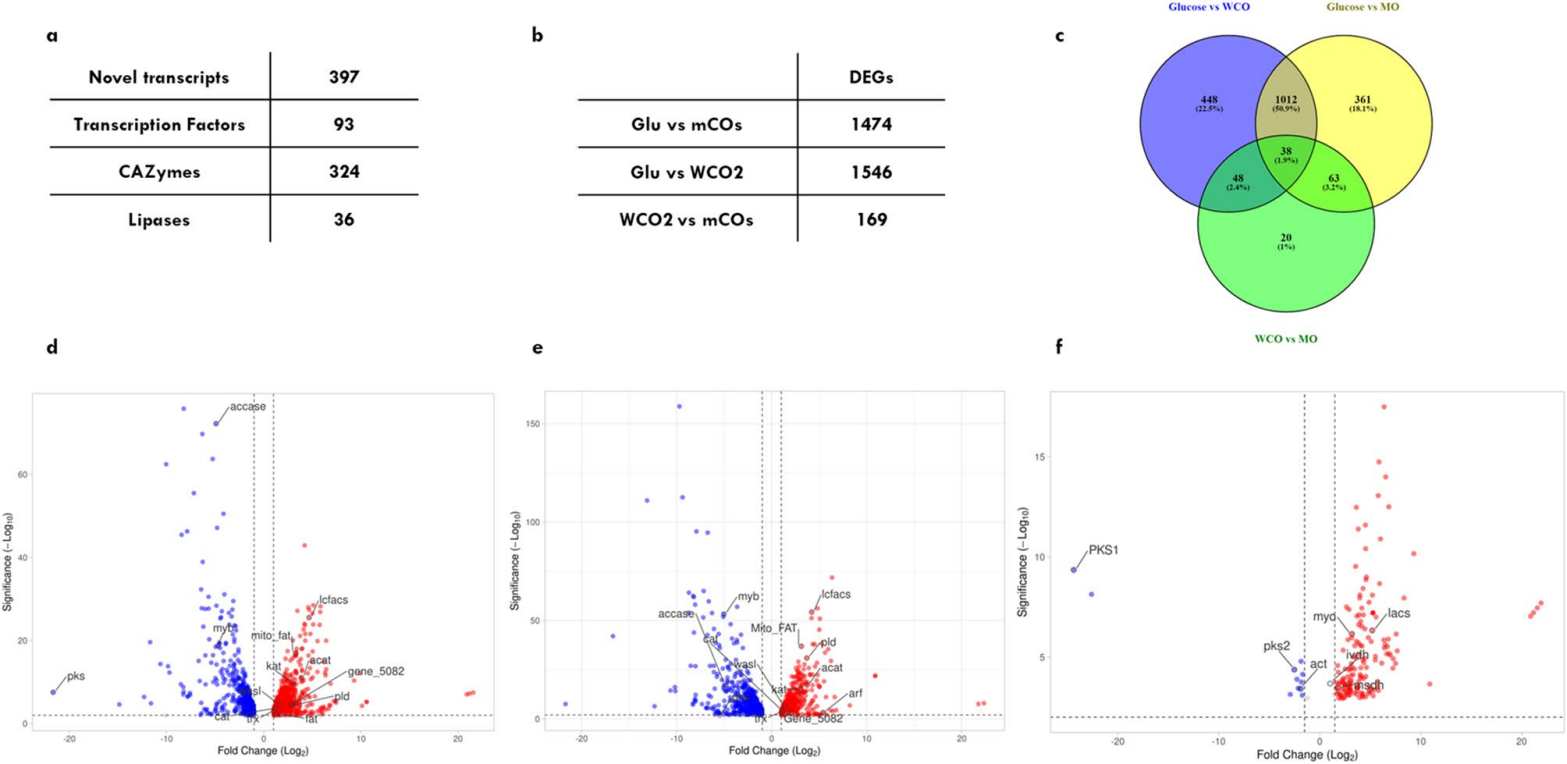
Transcriptomic analysis of *S. limacinum* SR21 in the presence of glucose, WCO2 and mCOS. **(a)** Table for different categories of transcripts annotated using computational tools. **(b)** Differentially expresses genes (DEGs) observed for varying conditions. **(c)** Venny diagram depicting overlap between WCO2 and mCOs. **(d)** Volcano plot highlighting few significant differentially expressed genes with (p-value < 0.05) and log2 fold-chain = > 1.5 for Glucose vs. WCO2; **(e)** Glu vs. mCOs; **(f)** and WCO2 vs. mCOs

To trace the metabolic route for assimilation and conversion of oil substrates, SR21 was cultivated in 20 g L^− 1^ of mCOs and WCO2 for 72 h and compared with cells cultivated in glucose. Transcriptomic analysis using the DNBseq platform generated 4.29 Gb bases per sample. The average mapping ratio with the reference genome was 97.24%. In the transcriptome dataset, 7129 genes were identified which include 397 novel coding transcripts (Fig. 4a) without any known features, and 589 long noncoding RNA. As depicted in Fig. 4b, compared to glucose, 1546 transcripts were differentially expressed in WCO2 (p-value < 0.05) whereas, 1474 were found for mCOs (p-value < 0.05). The volcano plot represented in Fig. 4d, e and f highlights some of the key transcripts differentially regulated in the presence of glucose, WCO2 and mCOs.

### Metabolic difference between WCO2 and mCOs

In contrast to the higher number of DEGs observed for glucose (Glu) vs. WCO2/mCOs, WCO2 vs. mCOs have only a few significant DEGs (Fig. 4c) suggesting the metabolic state of the cells cultivated in these two oils to be similar. Among 169 DEGs, only 12 transcripts were found to be significantly abundant in WCO2. As shown in Fig. 4f, WCO2 was found to have upregulation of genes comprising PKS clusters (*schi_nov_149* and *schi_nov_130*). The upregulated PKS in WCO2 suggests that SR21 was able to convert substrate FAs into intracellular DHA. As discussed previously, mCOs composition has fish oil-derived DHA, thereby the slightly higher intracellular DHA content might be from unutilized oil substrate.

Cells cultivated in WCO2 were found to have higher expression of the gene coding for palmitoyltransferase (*schi_0951*) and mitochondrial acylcarnitine transporter (*act*; *schi_5874*) which are involved in the transport of FA from the cytosol to the mitochondrial matrix, thereby enabling complete β-oxidation for the generation of acetyl-CoA [62, 63]. Whereas, in mCOs, genes coding for enoyl-CoA hydratase (*schi_3621*), isovaleryl-CoA dehydrogenase (*ivdh*; *schi_1991* and *schi_0625*) and methylmalonate semialdehyde (*msdh*; *schi_1866*) were upregulated, suggesting enhanced leucine degradation thereby generating acetyl-CoA. This suggests that the two HS trigger the utilization of different pathways for the generation of acetyl-CoA, required for energy generation in glucose-deprived conditions. Further, transcripts for several cytoskeletal elements such as myosin *(myo; schi_3067)* were found to be abundant in the case of mCOs. Type I myosin and WASL-interacting protein were found to be involved in clathrin-mediated endocytosis in microbial cells [64, 65]. Cells cultivated in mCOs reflect a higher abundance of transcripts for these genes (*myosin*I – s*chi_3067*; l*acs – schi_0997*) suggesting enhanced uptake of mCOs as compared to WCO2.

### Secretory lipases initiate the breakdown of TAG moieties in HS

Hydrophobic substrates (WCO2 and mCOs) are majorly composed of TAG moieties (Supplementary_file 1: Fig. 2), however, there is no evidence of uptake of such large molecular weight hydrophobic compounds in bacteria and eukaryotic microbes such as yeast [66]. In order to metabolize oil as a sole carbon substrate, thraustochytrid must degrade these TAG molecules to free fatty acids by secreting lipases in the extracellular environment [67]. The eukaryotic model yeast *Saccharomyces cerevisiae* is not known to secrete such lipases and thus is unable to catabolize oil substrates. These lipases contain a characteristic α/β hydrolase fold and can be further classified according to their sequence, structure and substrate specificity [68].

**Fig. 5:**
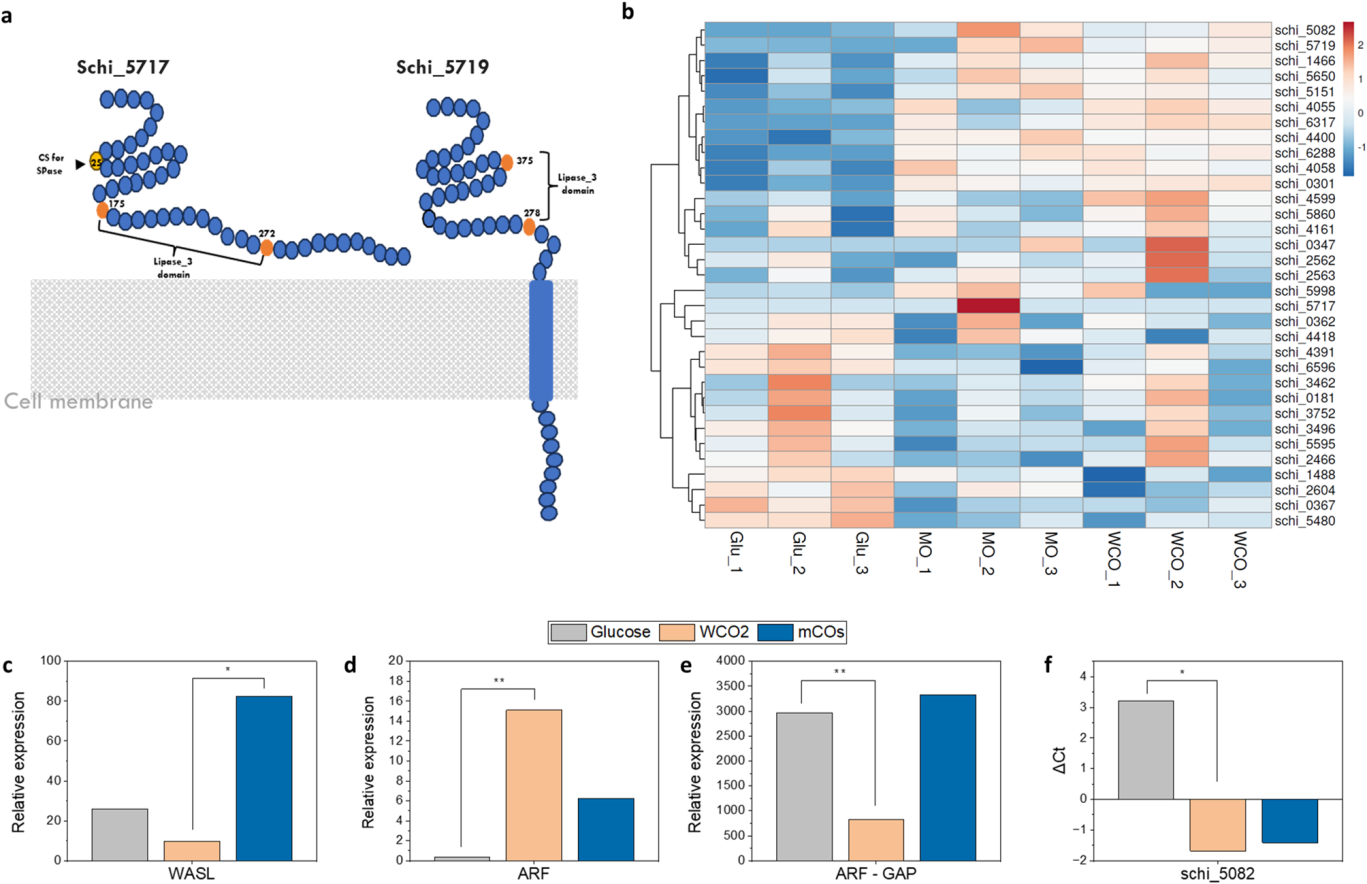
Secretory lipases and endocytosis allow oil utilization in SR21 **(a)** Schematic illustration for lipases (secretory and membrane-anchored) identified in S. limacinum SR21. Different features such as cleavage site (CS) for signal peptidase, lipase_3 domain and transmembrane domain is predicted using computational tools and highlighted; **(b)** Heatmap depicting the relative expression for all the lipases in SR21, the transcripts are clustered on the basis of correlation; Relative expression of **(c)** WASL-interacting protein; **(d)** ARF and **(e)** ARF-GAP, highlighting upregulated endocytosis in the presence of HS. Graph is plotted with transcript abundance (FPKM) of three biological replicates; **(f)** Relative expression of cytoplasmic TAG lipase (schi_5082) quantified using RT-PCR

In the current study, 36 lipases were identified that contain α/β hydrolase and belong to the lipid catabolic process using domain and GO analysis [69–71]. The majority of the lipases were found to contain either fungus-like Lipase 3 or patatin-like phospholipase domain. Localization prediction using DeepLOC 2.0 found schi_5719, schi_5480 and schi_5717 to be extracellular whereas only schi_5719 was predicted to be secretory by Wolfpsort (Supplementary_file 2). Proteins schi_ 5719 and schi_5650 were predicted to possess secretory signal peptides (Sec/SPI). Recently, a thraustochytrid-specific secretory lipase was identified for *A. limacinum*, which was predicted to be secretory but does not contain any secretory signal [11]. However, this protein (schi_5717) was found to have a transmembrane residue as identified using TMHMM 2.0. This reflects that schi_5717 might belong to the cell-wall anchored lipase category as reported for yeasts whereas schi_5719 is the major secretory lipase involved in the catabolism of TAG in oil substrates (Fig. 5a).

The heatmap displayed in Fig. 5b, illustrates the differential abundances of transcripts encoding for various lipases identified for SR21 in the presence of Glu, WCO2 and mCOs. The major secretory lipase (*schi_5719*) was found to be relatively abundant in WCO2 and mCOs as compared to Glu. However, the cell-wall anchored lipase (*schi_5717*) was found to be relatively similar in all three compared conditions. This suggests that upregulation of *schi_5719* allows SR21 to efficiently hydrolyze TAG into free fatty acids which are further transported to cytoplasm for degradation. Significant upregulation of lipase_3 containing protein schi_5082 was observed in both HS (Fig. 5b), which is also validated via RT-PCR (Fig. 5f). This protein was predicted to be cytosolic and lacks both secretory signal and transmembrane residue. However, no significant similarity was found with other annotated lipases present in the NR database and further research is required to assign functionality to this cytoplasmic lipase. The upregulation of cytoplasmic TAG lipase also reflects that certain TAG molecules are internalized by the plasma membrane and are then cleaved into fatty acids. Compared to FAs, TAG moieties have higher hydrophobicity as they lack –COOH residue, however, the import of TAG via passive diffusion has not been reported till date. Various other lipases belonging to the Phospholipase D family were found to be abundant in WCO2 and mCOs (1466, 5650, 6317, 0301) which resulted in efficient degradation of phospholipids in the substrate.

#### Endocytosis coupled with passive diffusion allows the import of HS

The released free fatty acids after hydrolysis by lipases should traverse the cell membrane in order to be metabolized as an energy source. In *S. cerevisiae*, import occurs via Fat1 protein, which is an ortholog of mammalian fatty acid transport protein [72]. However, this protein-mediated transport is not conserved among yeast species, for instance, a homologue of Fat1 in *Y. lipolytica* was found to be essential for its auxotrophic growth on FAs [73]. Jacquier et al. found that yeast mutants deficient in a protein kinase (*Δypk1*) failed to grow on fatty acid and established a model for endocytosis-mediated uptake of exogenous FAs [19]. However, the possible route for the uptake of FAs in thraustochytrids is not even hypothesized.

We identified several fatty acid transporters (TCDB – 4.C.1.1.1), similar to murine fatty acid transport protein 4 (FatP4) in SR21, however, none of them were predicted to be localized at the plasma membrane (Supplementary_file 3). Whereas we found upregulation of transcripts such as WASL interacting protein (schi_4668) (Fig. 5c) and neural Wiskott-Aldrich syndrome protein (N-WASP) in HS, which is involved in receptor-mediated endocytosis [74]. The ADP-ribosylation factor (ARF) is the role player in vesicle formation and thereby regulates endocytosis in eukaryotes [75]. This GTPase enzyme is active when bound with GTP and is inactivated by the action of the enzyme ARF-GTPase-activating protein (ARF-GAP) [76]. Cells cultivated in the presence of HS displayed a higher abundance of ARF (*schi_6223*) (Fig. 5d), whereas the ARF-GAP was downregulated (*schi_5057*) (Fig. 5e), reflecting active vesicle budding in the presence of oil. Apart from its role in vesicle formation, Arf proteins are also known to activate phospholipase D [77], and thereby modulate membrane lipid composition. PLD was found to be upregulated in HS, allowing the conversion of phosphatidylcholine (PC) to phosphatidic acid (PA), a lipid that favors membranes with negative curvature and thus can facilitate both membrane fission and fusion [77]. Transcriptomic data also highlighted differential expression of several cytoskeletal elements such as myosin I which is known to be involved in endocytosis [65]. Thus, the lack of membrane FA transporters and upregulation of proteins such as Arf, Pld and WASL suggest that thraustochytrids rely on passive diffusion and endocytosis for the uptake of exogenous FAs (Fig. 4a and b).

### Coupling of peroxisomal and mitochondrial β-oxidation to generate acetyl-CoA

**Fig. 6:**
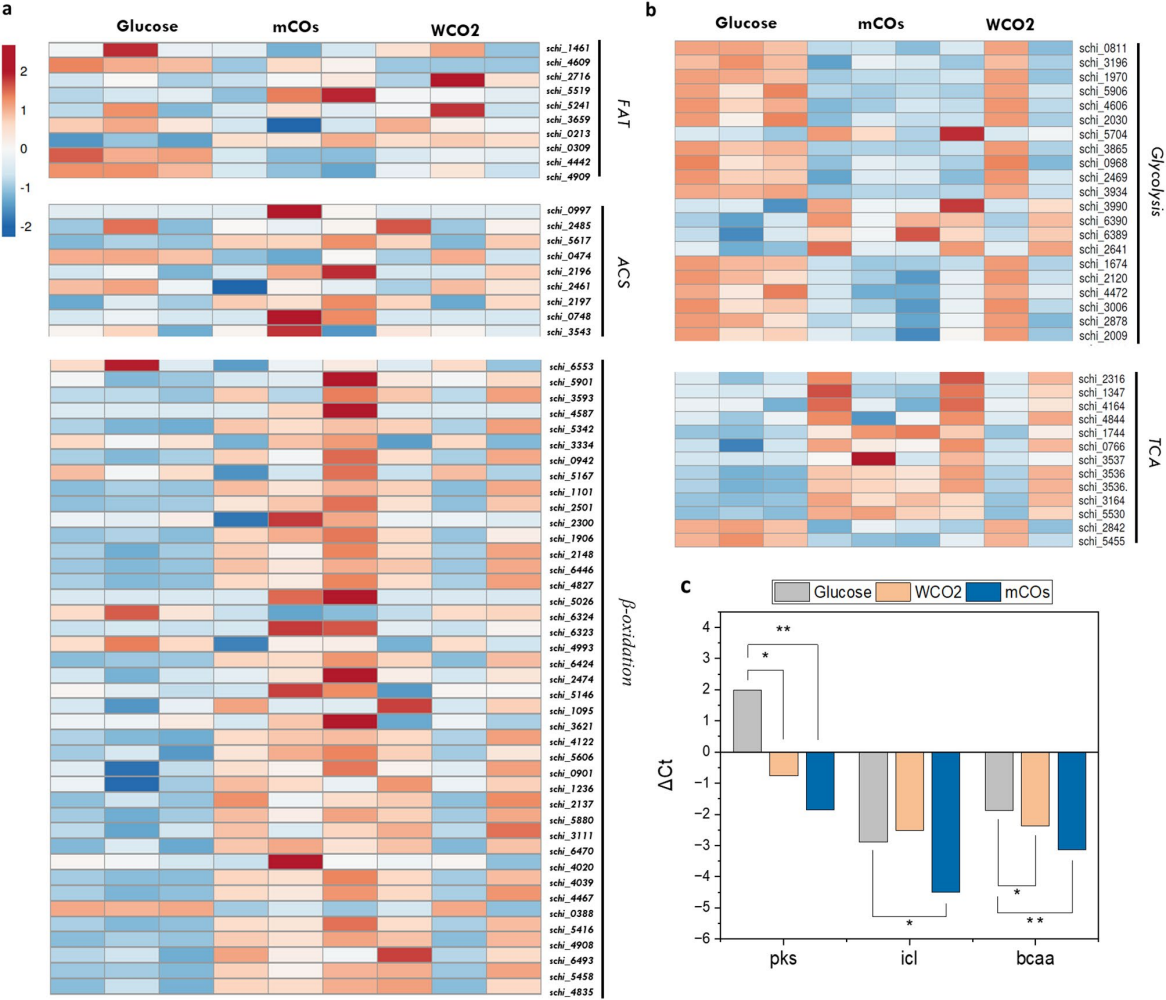
Differential expression of genes participating in various pathways **(a)** Lipid catabolism; **(b)** carbon metabolism depicted in the form of heatmap; **(c)** RT-PCR validation of significant genes (*pks: polyketide synthase, icl: isocitrate lyase, bcc: branched chain amino acid*). Data represents ΔC_t_ value normalized using 18 S and actin as internal control and is mean of biological triplicates

As discussed previously, the uptake of fatty acids is followed by its activation by ACS, that convert hydrophobic moieties into soluble acyl-CoA thioesters, directing it further in the cytoplasmic organelles [78]. In *S. cerevisae*, the Fat1 transporter itself contains the ACS in the C-terminus, thereby involved in both the uptake and activation of FAs [79]. Two additional ACS are reported for *S. cerevisae*, i.e., Faa1 and Faa4 which interact with Fat1 to form a complex [80]. Genomic data for *Arabidopsis* encode nine ACS whereas the marine diatom *Phaeodactylum tricornutum* possesses five ACS [81, 82]. In SR21, nine putative ACS have been identified, which are distributed in the cytoplasm, peroxisome, cell membrane and ER (Supplementary_file 4). In comparison to Glu, several ACS encoding transcripts such as *schi_0997, schi_5617 and schi_2197* (Fig. 6a) were found to be significantly abundant in the cells cultivated in HS. As discussed previously, the upregulated transcripts might be specific for C16 FAs, and are thus involved in their activation and assimilation, directing them towards β-oxidation.

Thraustochytrids are known to employ both peroxisomal and mitochondrial β-oxidation for the degradation of FAs [83, 84]. The peroxisomal and mitochondrial localized FAT were found to be upregulated in SR21 cultivated in WCO2 and mCOs (Fig. 6a). The transcripts for enzymes participating in the oxidation of FAs in both mitochondria and peroxisome were also found to be upregulated (Fig. 6a). The enzyme acyl-CoA oxidase (ACOX) catalyzes the first step in peroxisomal β-oxidation, whereas in mitochondria acyl-CoA dehydrogenases (ACAD) are responsible for the removal of hydrogen atoms. Watanabe et al. generated *Δacox* mutants of *Aurantiochytrium* sp. and found enhanced DHA accumulation [85]. The enzyme catalyzing the final step in FA oxidation; ketoacyl-CoA thiolase (KAT) was found to be significantly higher in the presence of HS (Fig. 6a). The mechanistic activity of KAT results in the removal of two carbon atoms and the generated (n-2) FA is processed again through the same oxidation cycle till the formation of acetoacetyl-CoA. Short-chain FAs are transferred from peroxisome to mitochondria for complete oxidation. Mitochondrial acetyl-CoA acetyltransferase catalyzes the final reversible reaction to generate two molecules of acetyl-CoA and was found to be significantly higher in the presence of HS [86].

Another key player in both peroxisomal and mitochondrial oxidation is carnitine, which allows the translocation of FA intermediates from cytosol via the carnitine cycle [87]. Carnitine palmitoyltransferase is a mitochondrial outer membrane protein that attaches carnitine moiety to fatty acyl-CoA generating acylcarnitines. These fatty acylcarnitines are then translocated into the mitochondrial matrix and are acted upon by carnitine acetyltransferase to generate carnitine and fatty acyl-CoA, which further undergoes mitochondrial β-oxidation [88]. Along with mitochondrial carnitine acyltransferases, SR21 also possesses certain peroxisomal CAT which might aid in the export of FA oxidation products to the cytosol or mitochondria. Figure 6a displays significant upregulation of both carnitine palmitoyltransferase and carnitine acetyltransferase in the presence of HS. Thus, the transcriptomic data suggests that in the presence of HS, SR21 cells efficiently catabolize FAs in the substrate by upregulating both mitochondrial and peroxisomal β-oxidation to generate acetyl-CoA.

### Channeling of acetyl-CoA among lipid biosynthesis, TCA and amino acid metabolism

In the presence of HS, genes participating in hexose metabolism i.e., glucose, were found to be significantly downregulated (Fig. 6b). Similarly, the expression of genes coding for enzymes participating in the pentose phosphate pathway (PPP) was significantly lower in the cells cultivated in oil. The two NADPH generating enzymes of PPP; glucose-6-phosphate dehydrogenase (*schi_3912*, and *schi_4427*) and gluconate-6-phosphate dehydrogenase (*schi_5078*) were found to be downregulated in the presence of WCO2 and mCOs as compared to glucose, which results in lower NADPH required for fatty acid biosynthesis. In such cases, NADPH can be regenerated using two anaplerotic enzymes isocitrate dehydrogenase and malic enzyme. In the current study, isocitrate dehydrogenase (*schi_3120*) was found to be significantly upregulated in the presence of both HS (Supplementary_file: 1, Fig. 3). Fatty acid oxidation is the sole pathway for the generation of acetyl-CoA in the presence of HS, thus expression of pyruvate kinase and pyruvate dehydrogenase was significantly lower in comparison to glucose. The acetyl-CoA generated has several anabolic fates; it can be carboxylated to form malonyl-CoA and enter the lipid pathway, or it can act as a precursor for terpenoids and enter the mevalonate pathway. Citrate synthase utilizes the acetyl-CoA molecule and converts it into citrate, thereby tagging it for the TCA cycle. In the presence of WCO2 and mCOs, citrate synthase and other TCA cycle enzymes were found to be significantly higher (Fig. 6b). We found upregulation of succinate dehydrogenase enzyme in the presence of HS, that links TCA to oxidative phosphorylation and provides electrons to quinones leading to ATP generation (Fig. 6b). This further provides the metabolic insights for the previously observed higher biomass for SR21 grown in HS. Lipids have been known to be a better energy source than glucose, i.e., palmitate yields 4.1 high energy phosphate bonds per mol of O_2_, which is relatively higher than glucose [89].

In the presence of nutritional stress, the acetyl-CoA generated is used for anabolic processes via glyoxylate shunt. Isocitrate lyase (ICL) converts isocitrate to glyoxylate and succinate and the glyoxylate generated is further utilized by malate synthase (MS) for the formation of malate which then is redirected towards gluconeogenesis [90]. Cells cultivated in HS have higher abundances of both *icl* (*schi_3536)* and *ms*, suggesting an efficient glyoxylate cycle for the biosynthesis of other molecules. Figure 6c also highlights the transcript abundance for *icl*, validated using RT-PCR. Further, the branched-chain amino acid metabolism (Val, Leu and Ile) was also found to be significantly upregulated in the presence of HS along with the synthesis of serine and glycine. The degradation of branched-chain amino acid further leads to the production of acetyl-CoA and other TCA intermediates, thereby providing electrons for enhanced ATP generation.

**Fig. 7:**
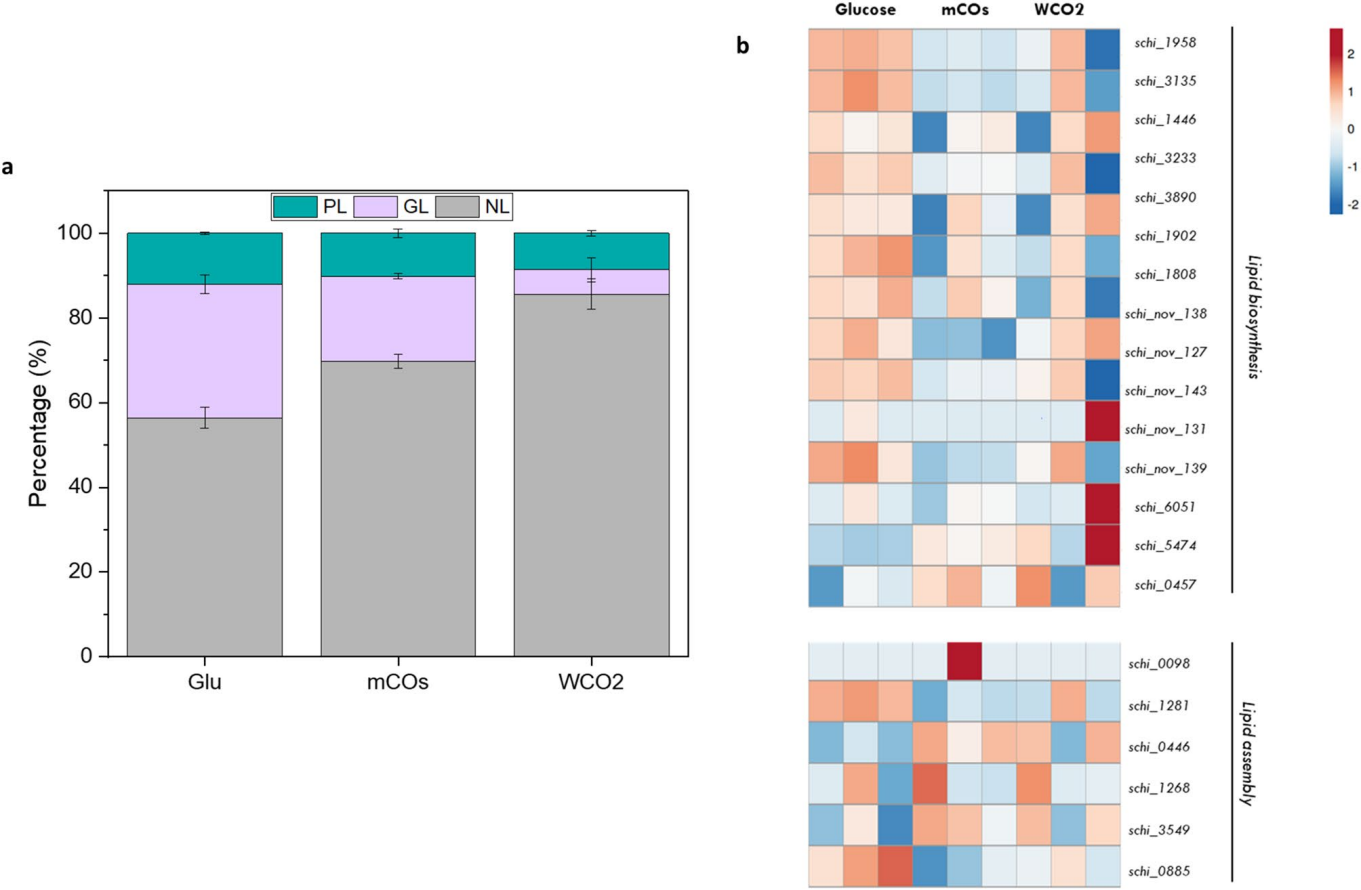
Effect of oil substrates on lipid synthesis in SR21 **(a)** Lipid fractionation of SR21 intracellular lipids cultivated in 20 g L^− 1^ glucose, mCOs and WCO2. The lipids were separated using a silica-column according to their neutral lipid (NL), glycolipid (GL) and phospholipid (PL) content; **(b)** Heatmap for relative expression of transcripts involved in lipid synthesis and assembly

Intracellular lipid accumulation is initiated by carboxylation of the acetyl-CoA molecule catalyzed by acetyl-CoA carboxylase *(schi_1958)*, which was relatively low in HS as compared to glucose (Fig. 7b). Thraustochytrids are known to possess an alternative PKS-dependent pathway for the biosynthesis of long-chain PUFAs. SR21 is predicted to contain six ORFs corresponding to PKS clusters. Interestingly, all the transcripts were found to be significantly lower in the presence of HS *(schi_nov_138, schi_nov_127, schi_nov_143, schi_nov_131, schi_nov_139, schi_6051).* Downregulation of FA synthesis genes along with lower DHA (Fig. 2a) in the presence of HS can be inferred as enhanced acetyl-CoA partitioning towards essential cell constituents such as amino acids and nucleotides than lipid molecules. The substantial conversion of HS into DHA can be attributed to basal expression of PKS clusters. The accumulated pools of unsaturated fatty acids C16:1 and C16:2 can be justified by the upregulated desaturase (*schi_0457)* (Fig. 7b) in the presence of HS, while they were not observed for glucose.

### Effect of HS on intracellular lipid partitioning

Intracellular lipid composition is species-dependent and varies significantly according to the cultivation conditions. To pinpoint the differences among the lipid classes of the intracellular lipids synthesized via the “*de novo*” and “*ex novo*” lipid synthesis pathway, a fractionation method was used to separate the lipids into neutral lipid (NL), glycolipid (GL) and phospholipid (PL). As shown in Fig. 7a. the major lipid fraction corresponds to NLs, with lower levels for glucose (56.5 ± 2.5%), while they increased for mCOs (69.6 ± 1.6%) and maximized for WCO2 (85.7 ± 3.6%). As expected, GLs and PLs followed the opposite trend, with fractions belonging to glucose and mCOs having GLs (31.6 ± 2.2 and 20.1% ± 0.6% respectively) as the predominant type of lipids, while WCO2 has slightly higher PL content as compared to GL.

Microbial oil is comprised of free fatty acids (FFAs) with different saturation levels and different lipid classes (polar and non-polar) [91]. Neutral lipids do not contain any polar functional groups and mainly consist of TAGs, as well as monoacylglycerols (MAGs), diacylglycerols (DAGs) and sterols [92]. TAGs are used as reserve lipids for metabolic energy purposes and are the main component of cytosolic lipid droplets. Neutral lipid TAGs consist mainly of SFAs and MUFAs and to a lesser extent of PUFAs since these are mostly prominent in polar lipids [93]. The latter include phospholipids and glycolipids which are highly unsaturated long carbon chain lipids and main components of the cell membrane. Overall, the lipids synthesized during the “*de novo*” pathway contained higher polar lipids (43.6% total PLs and GLs), which were 1.4-fold and 2.1-fold higher than mCOs and WCO2. That is an anticipated result, as DHA-rich PUFAs are mainly found in polar lipids and were also measured 1.3-fold and 1.7-fold higher in the lipids synthesized during “*de novo*” pathway compared to the “*ex novo*”.

The increased content of NLs for the HS is associated with an elevated TAG content since TAGs have been reported to be the main component of NLs for thraustochytrids [94]. Thin layer chromatography (TLC) results (Supplementary_file 1: Fig. 2) also verified a trend where higher TAGs were present in the intracellular lipids produced in WCO2 media, followed by mCOs and glucose. *De novo* TAG assembly occurs via the Kennedy pathway in ER of the eukaryotic cell and involves the participation of three enzymes glycerol 3-phosphate acyltransferase (GPAT), lysophosphatidic acid (LPAT) and diacylglycerol acyltransferase (DGAT). The committed step of the pathway is the conversion of diacylglycerol (DAG) to TAG moieties, which is mediated by DGAT. As illustrated in Fig. 7b, in comparison to glucose, the ER localized GPAT was found to be not differentially expressed in WCO2 (p-value = 0.08) and mCOs (p-value = 0.27). Similarly, LPAT expression was found to be unaltered in SR21 cells cultivated in glucose and HS. In the green microalga *C. reinhardtii*, both GPAT and LPAT were found to be not important for TAG assembly [95]. However, *Aurantiochytrium* sp. cultivated in cold stress (5 ^o^C) for TAG accumulation was found to have 1.2 and 12-fold upregulation of GPAT and LPAT respectively [96]. Three isoforms of DGAT were found for SR21 i.e., *schi_1281*, *schi_0446* and *schi_1268*. Among these three; *schi_1281* was downregulated, *schi_0446* was upregulated whereas *schi_1268* was found to have no differential expression in both HS (Fig. 7b). In *Aurantiochytrium* sp., three DGAT isoforms were found to be specific for the transfer of acyl chains with different saturation levels. The DGAT2C was responsible for adding PUFAs, whereas DGAT2A and DGAT2D add SFAs to the TAG moieties [97]. Thus, the varying expression pattern for different DGATs in SR21 might be responsible for adding different fatty acids to TAG molecules.

Another acyl-CoA independent route for the synthesis of TAG is through phospholipids remodeling. The enzyme phospholipid: diacylglycerol acyltransferase (PDAT) is involved in transferring acyl-group from phospholipids (PL) to diacylglycerol (DAG) to form TAG. However, this enzyme was not significantly varied among glucose and HS (Fig. 7b), suggesting that DGAT (*schi_0446*) was the only one responsible for TAG synthesis in HS. The relative abundance of phospholipase C and diacylglycerol diphosphate phosphatase (Fig. 7b) in SR21 cultivated in HS might contribute to the accumulated pool of DAG, which is further acted upon by DGAT for its conversion to TAG. Thus, SR21 employs a combination of Kennedy pathway and phospholipid remodeling for TAG accumulation in the presence of HS.

### *S. Limacinum* SR21 shifts its metabolism to cope with oxidative stress

**Fig. 8:**
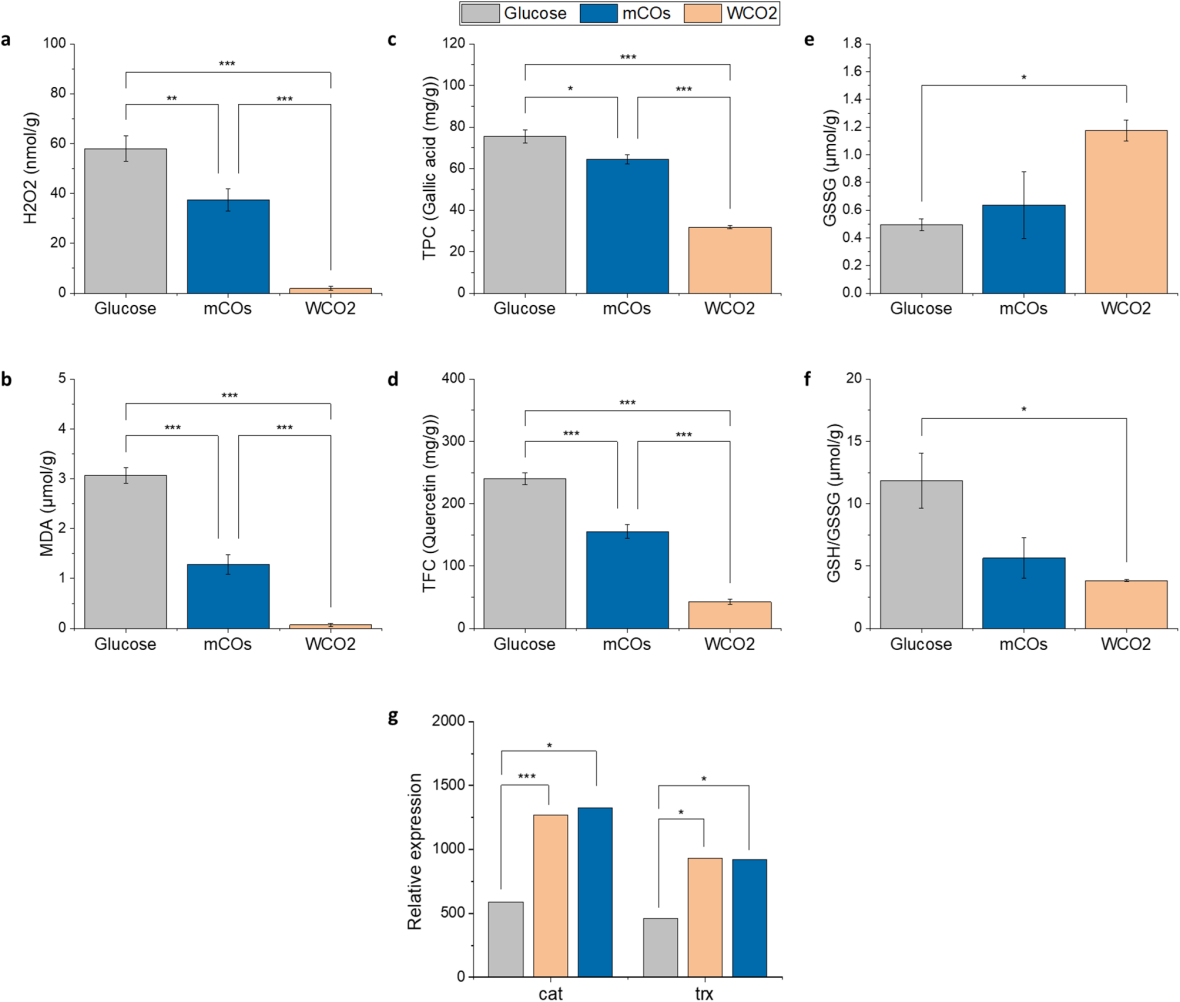
Determination of SR21 cell’s oxidative state using stress biomarkers and non-enzymatic antioxidants after 72 h. **(a)** Hydrogen peroxide (H_2_O_2_) estimation as a reactive oxygen species (ROS) generated during β-oxidation, expressed in nmol g^− 1^ dried biomass; **(b)** Lipid peroxidation in terms of the malondiadehyde (MDA) content expressed in µmol g^− 1^ dried biomass; **(c)** Total phenolic (TPC) content of methanolic cell extracts expressed in mg gallic acid g^− 1^ dried biomass; **(d)** Total flavonoid content (TFC) of methanolic cell extracts expressed in mg quercetin g^− 1^ dried biomass; **(e)** Oxidized glutathione (GSSG) levels expressed in µmol g^− 1^ dried biomass; **(f)** Ratio of (GSH/GSSG) and **(g)** Relative expression of mitochondrial catalase (cat) and thioredoxin reductase (trx). The asterisks denote statistical significance * (p < = 0.05), ** (p < = 0.01), *** (p < = 0.001)

In order to investigate the effect of oxidative stress imposed by HS, several non-enzymatic oxidative stress markers were quantified. The oxidative status of the cells was further evaluated by correlating these data with the differential abundance of enzymatic machinery obtained through transcriptomic analysis. Under stress conditions, there is an imbalance between the antioxidant defense system of the cells and the reactive oxygen species (ROS) production, leading to an accumulation of certain molecules, such as superoxide anion radicals (O_2_^−^), hydrogen peroxide (H_2_O_2_), hydroxyl radicals (•OH) and singlet oxygen (^1^O_2_) [98, 99]. Figure 8 depicts a noticeable trend, where SR21 grown on glucose had higher levels of H_2_O_2_, malondialdehyde (MDA) and antioxidant biomarkers, followed by mCOs and WCO2.

Malondialdehyde (MDA) and 4-hydroxy-2-nonenal (HNE) are aldehydes that are produced as a result of lipid peroxidation [100]. In SR21, the MDA level was 3.1 µmol g^− 1^ in glucose-grown cells, which was 2.4-fold and 44-fold higher than that of mCOs and WCO2 (Fig. 8b). The higher levels of lipid peroxidation observed in the presence of glucose can be associated with the elevated PUFA content present in the intracellular lipids, especially FAs of more than 20 carbon atoms, whereas the intracellular lipid composition of cells grown in HS is rich in C18 PUFAs. Also, TLC results (Supplementary_file 1: Fig. 2) indicated higher levels of free fatty acids (FFAs) in the intracellular lipids for glucose, which are more easily oxidized than TAGs.

Notably, the lowest levels of biomarkers such as H_2_O_2_ was observed for HS (Fig. 8a), particularly WCO2, highlighting the ability of the strain to adapt remarkably well in highly saline and oily environments. This is also verified by the increased biomass production that is achieved after cultivation on HS, compared to glucose media. The accumulated pool of H_2_O_2_ is degraded by several enzymatic machinery such as catalase (CAT), glutathione peroxidase (GPX) and thioredoxin reductase (TRXR) [101]. GPX reduces H_2_O_2_ by using GSH or thioredoxin (TRX) as electron donors, thereby converting GSH to its oxidized form (GSSG) and forming H_2_O [102, 103]. Figure 8e and f, depict the amounts of GSSG for SR21, as well as the ratio of GSH/GSSG. In the presence of glucose, the H_2_O_2_ content was relatively higher which induced GPX to reduce GSSG to GSH, and thus higher levels of GSH/GSSG were observed. The transcriptome data further validates the upregulation of two isoforms of GPX (p-value not significant). Although, the slight upregulation of TRXR (p-value < 0.05) in the presence of HS was observed, suggesting that thioredoxin system is preferred over glutathione.

Transcriptomic data also highlights that in the presence of HS, the expression of superoxide dismutase (SOD) was relatively similar to cells cultivated in glucose, whereas mitochondrial catalase was found to be upregulated (Fig. 8g). The abundant transcript for catalase might be responsible for the successful mitigation of ROS generated from both fatty acid oxidation and upregulated oxidative phosphorylation in the presence of HS.

Further, the total phenolic content (TPC) and total flavonoid content (TFC) that act as non-enzymatic antioxidants were observed to be higher in glucose, with 1.5-fold and 5.6-fold higher TFC (240 mg QE g^− 1^) and 1.2-fold and 2.4-fold higher TPC (76 mg GA g^− 1^), in comparison to HS (Fig. 8c and d). Phenolic compounds have very good reduction potential due to the electron donating activity of the “acidic” phenolic hydroxyl group and much lower values than the reduction potentials of the oxygen radicals, therefore can scavenge harmful reactive oxygen intermediates successfully [103]. This diverse category involves a wide range of compounds, such as flavonoids, phenolic acids and other polyphenols which are related to the antioxidant activity of microorganisms [104]. Certain studies tried to correlate the antioxidant performance of thraustochytrids with their phenolic and flavonoid content. In particular, certain cases found the phenolic content to be responsible for the antioxidant capacity of the cell extracts [58, 105], while others proved the flavonoid content significantly correlated [106]. Thus, SR21 cultivated in HS seems to maintain the cellular redox-balance efficiently with lower H_2_O_2_ and other antioxidants as compared to glucose. Additionally, in order to cope with compartmentalized oxidative load in mitochondria, SR21 upregulates catalase which allows the thraustochytrid to grow efficiently in a hydrophobic environment.

## Conclusions

**Fig. 9:**
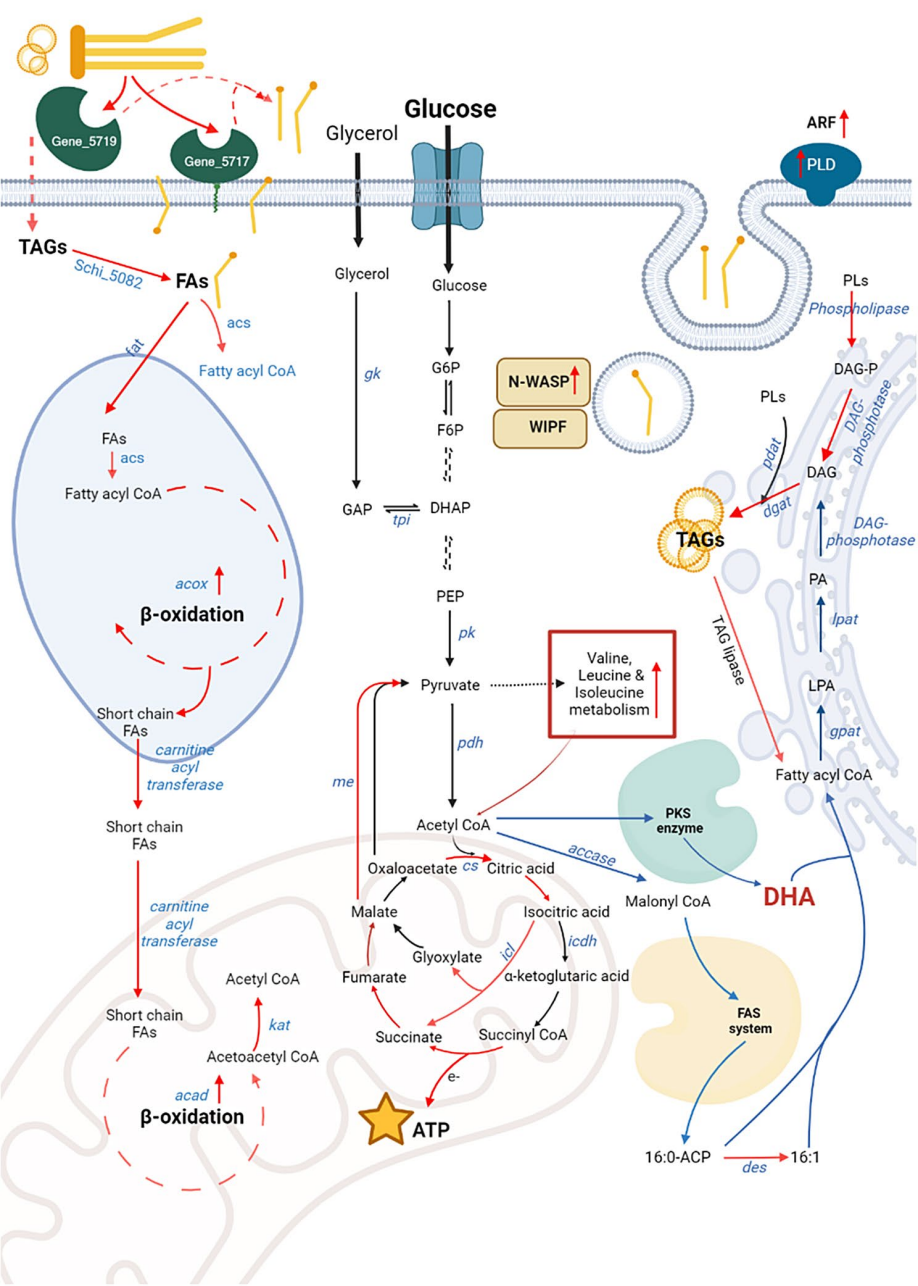
Schematic illustration for hydrophobic waste oil assimilation in thraustochytrid SR21. (The red lines highlight the biosynthetic steps upregulated in the presence of HS, black line denotes the route followed by glucose alone, whereas blue line denotes the process common in both “*de novo”* and “*ex-novo*” cultivation) created with Biorender.com. *gk: glycerol kinase, tpi: triose phosphate isomerase, accase: acetyl-CoA carboxylase, pk: pyruvate kinase,, pdh: pyruvate dehydrogenase, pdc: icl: isocitrate lyase, icdh: isocitrate dehydrogenase, me: malic enzyme, des: desaturases*; *pdat: phospholipid: diacylglycerol acyltransferase, arf: ADP-ribosylation factor, fat: fatty acid transporter, acs: acyl-CoA synthetase, acox: acyl-CoA oxidase, acad: acyl-CoA dehydrogenases, kat: ketoacyl CoA-thiolase, N-WASP: Wiskott-Aldrich syndrome protein* PL: phospholipids, G6P: glucose 6-phosphate, F6P: fructose-6-phosphate, GAP: glyceraldehyde-3-phosphate, DHAP: dihydroxyacetone phosphate, PEP: phosphoenol pyruvate, LPA: lysophosphatidic acid, PA: phosphatidic acid, DAG: diacylglycerol, TAG: triacylglycerol

This study provides the metabolic route for the assimilation of HS in SR21 (Fig. 9) and identifies the regulatory nodes that can be modulated to enhance the valorization of HS towards nutraceutical DHA. Expression of secretory, membrane-anchored and cellular lipases and incorporation of fatty acids via passive diffusion coupled with endocytosis suggest its similarity with the eukaryotic yeast species. However, the cellular physiology of the marine protist is unique as it employs both mitochondrial and peroxisomal β-oxidation for the complete oxidation of FAs and redirects the catabolic products of oil substrates towards both energy generation and intracellular DHA accumulation. Thus, we propose *S. limacinum* SR21 as an ideal candidate, with the potential for sustainable degradation and bio-transformation of waste streams into value-added products.

### Electronic supplementary material

Below is the link to the electronic supplementary material.

Supplementary Material 1

Supplementary Material 2

Supplementary Material 3

Supplementary Material 4

Supplementary Material 5

Supplementary Material 6

Supplementary Material 7

## Data Availability

Transcriptome data have been deposited in the Gene Expression Omnibus (https://www.ncbi.nlm.nih.gov/geo/) with Accession ID: GSE252403. All other relevant data are available from the authors upon reasonable request.
