# Supplementary material for: Transcriptomics aids in uncovering the metabolic shifts and molecular machinery of Schizochytrium limacinum during biotransformation of hydrophobic substrates to docosahexaenoic acid
Source: Microb Cell Fact. 2024 Apr 1;23:97. doi: 10.1186/s12934-024-02381-6 (PMC10983653; doi:10.1186/s12934-024-02381-6)
Supplement: Supplementary file 1 — Supplementary Material 1 [file 12934_2024_2381_MOESM1_ESM.docx]

Fig. 1: Batch cultivation of SR21 in 10-80 g L^-1^ mCOs. Growth associated parameters (substrate consumption (%), dry cell weight (DCW; g L^-1^)), lipid and DHA production (% and g L^-1^) are shown. The indicated values represent the mean and s.d of three biological replicates.


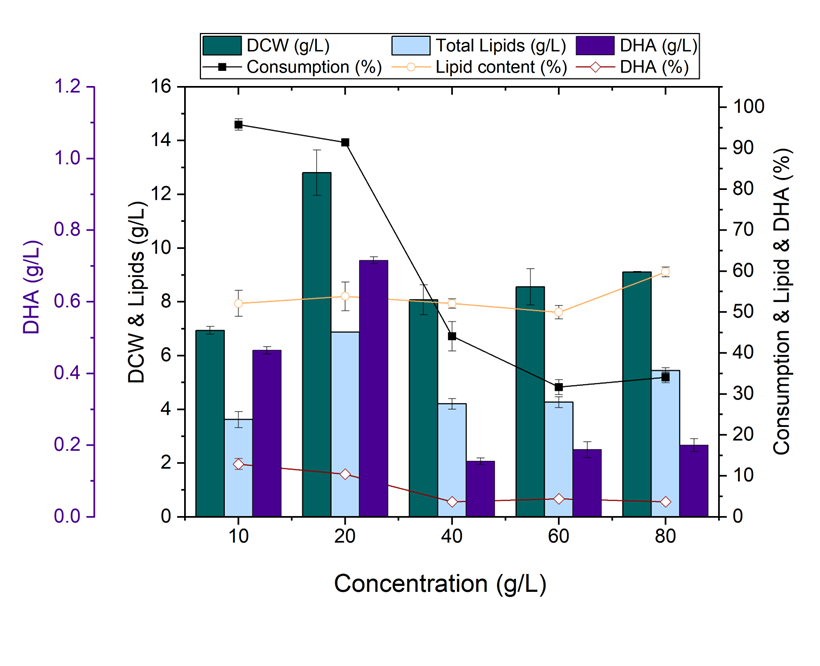


Fig. 2: Thin Layer Chromatography (TLC) of mCOs, WCO2 at 0 and 72 h and intracellular lipids from SR21 grown in glucose, mCOs and WCO2 after 72 h.


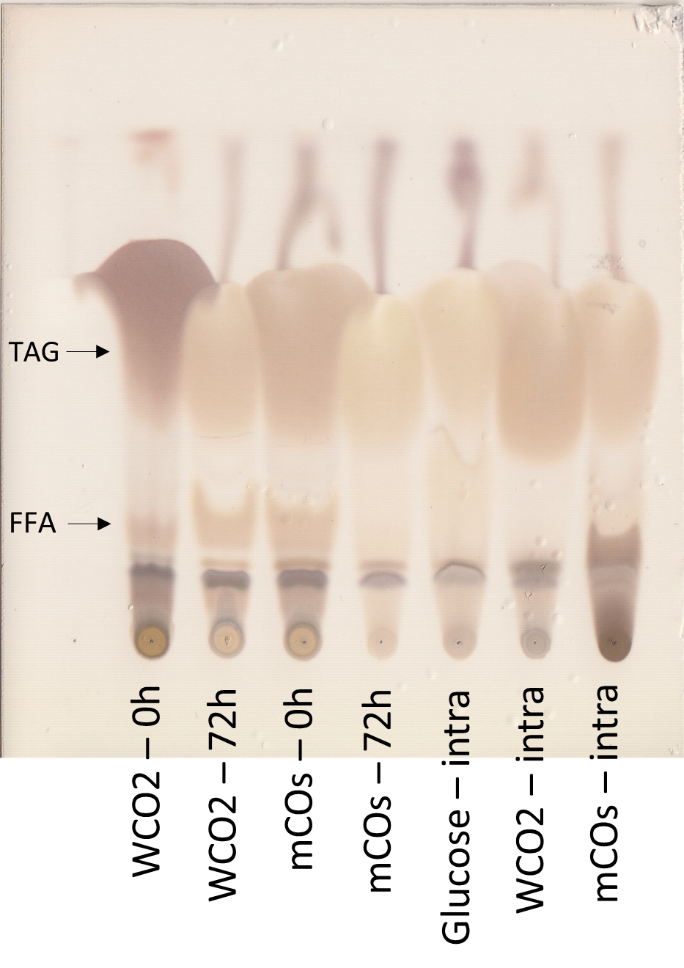


Figure 3: Transcript abundance for isocitrate dehydrogenase (*schi_3120)* in the presence of Glucose, WCO2 and mCOs.


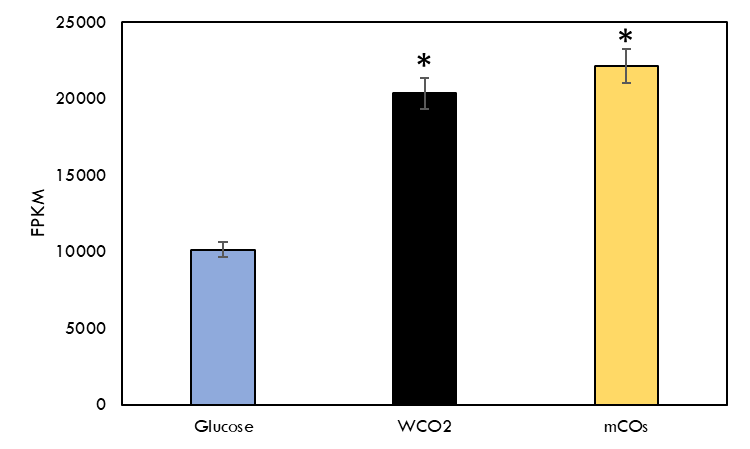


Table 1: Sequence for primers used for qPCR analysis.

| Gene name | Forward primer | Reverse primer |
| --- | --- | --- |
| 18S | AGCGAGTAGAGGAGGAAGAA | CCTCCGACTGTGCTTTAACT |
| *schi_5082 (*Lipase_3) | CGCCATGCTTGTACCAAATAC | CCTCGGGAATGCAAGTATGA |
| *pks* | GGTCAAGACCACCAAGGATAAG | TCGGAGTCCTCCATCTGAAA |
| *icl* | CACTCGCTTCAACGAGAAGA | TGCCATCCAGAGACGTAGA |
| *bcaa* | GAGGAGTTCTCTACCTCGAACT | CATCTGGTTGGTCACACTCTT |
